# Characterization in rabbits & nonhuman primates of the neutralizing antibody response elicited by DNA & protein vaccination with SIVmac251 & SIVsmE660

**DOI:** 10.1186/1742-4690-9-S2-P43

**Published:** 2012-09-13

**Authors:** VP Menon, S Whitney, J Francis, L Ajayi, N Petrovsky, D Montefiori, R Pal, K Vaniambadi

**Affiliations:** 1 Advanced Biosciences Laboratories, Rockville, MD, USA; 2 Flinders Medical Centre, Bedford Park,SA, Australia; 3 Dept of Surgery, Duke University Medical Center, Durham, NC, USA

## Background

Partial success of the human RV144 clinical trial underscored the importance of envelope antibody responses for an effective HIV-1 vaccine. Immunogenicity studies with SIV envelope proteins typically neutralize TCLA isolates. Properly folded trimeric envelope proteins delivered with appropriate adjuvants may successfully elicit antibodies with broad neutralization specificity.

## Methods

We evaluated immunogenicity of DNA and protein vaccines encoding SIVmac251 and SIVsmE660 gp145 in rabbits and rhesus macaques. DNA vaccines encoding wild type gp145 or mutated gp160 truncated at Q708 were used. Trimeric wildtype gp145 proteins, stably expressed and purified from 293T cells, were used with Advax delta inulin adjuvant to boost after DNA immunization. Macaques were electroporated with wild type DNA of both isolates followed by adjuvanted homologous protein boosts. Rabbits received DNA vaccine alone, delivered by electroporation. Neutralization assays were performed in TZM-bl cells with SIVmac251 and SIVsmE660 isolates that are partially resistant to neutralization.

## Results

Anti-envelope responses to SIV mac251gp145 and SIVsm660 gp145 were detected in macaque sera following DNA immunizations and response were enhanced significantly after adjuvanted homologous gp145 protein boost. At peak response, low to moderate neutralizing activity was observed against SIVmac251/M766, SIVsmE660-BR/CG7V and SIVmac251.30 clones. Immunization of rabbits with DNA encoding, either wild type or mutated envelope elicited strong antibody responses and sera neutralized both SIVmac251/766 and SIVsmE660-BR/CG7V to a limited extent, with a comparable response noted for both wild type and mutant envelope.

## Conclusion

Antibody response elicited by DNA prime/Advax adjuvanted protein boost vaccine with oligomeric envelopes from both SIVmac251 and SIVsmE660 neutralized SIVmac251 and SIVsmE660 isolates of partially resistant phenotype. These envelopes together with other antigens that elicit cellular responses will be tested against SIV challenge in future efficacy studies.

